# Identification of an Immune Gene-Associated Prognostic Signature and Its Association With a Poor Prognosis in Gastric Cancer Patients

**DOI:** 10.3389/fonc.2020.629909

**Published:** 2021-02-08

**Authors:** Xiaoqing Guan, Zhi-Yuan Xu, Runzhe Chen, Jiang-Jiang Qin, Xiang-Dong Cheng

**Affiliations:** ^1^Institute of Cancer and Basic Medicine, Chinese Academy of Sciences, Cancer Hospital of the University of Chinese Academy of Sciences, Zhejiang Cancer Hospital, Hangzhou, China; ^2^Department of Thoracic/Head and Neck Medical Oncology, The University of Texas MD Anderson Cancer Center, Houston, TX, United States

**Keywords:** gastric cancer, ssGSEA, CIBERSORT, tumor immunity, immune signature

## Abstract

The immune response plays a critical role in gastric cancer (GC) development, metastasis, and treatment. A better understanding of the tumor-immune system interactions in gastric cancer may provide promising diagnostic, prognostic, and therapeutic biomarkers for patients with this disease. In the present study, we aimed to identify a prognostic signature of GC through a comprehensive bioinformatics analysis on the tumor-immune interactions as well as the molecular characteristics. We firstly identified two immunophenotypes and immunological characteristics by employing multiple algorithms, such as the single sample Gene Sets Enrichment Analysis and Cell type Identification By Estimating Relative Subsets of RNA Transcripts. Next, we developed a six-immune-gene signature as a promising independent prognostic biomarker for GC using Lasso Cox regression and verified it *via* the external validation set and systematically correlated the immune signature with GC clinicopathologic features and genomic characteristics. Finally, a nomogram was successfully constructed based on the immune signature and clinical characteristics and showed a high potential for GC prognosis prediction. This study may shed light on the treatment strategies for GC patients from the perspective of immunology.

## Introduction

Despite the significant decreases in incidence and mortality during the last decades, gastric cancer (GC) remains the third leading cause of cancer-related deaths worldwide. There were over one million newly diagnosed GC patients and 783,000 deaths from this heterogeneous disease in 2018 (1). The GC incidence rates for men are twice as high as that of women. Besides, the GC incidence rates are generally low in Northern America and Northern Europe but markedly high in Eastern Asia (e.g., Japan, the Republic of Korea, and China) (2, 3). Histopathologic and epidemiologic features of GC vary widely between Asian and Western populations (4, 5). Also, the behavior and outcomes of patients with GC proved to be different comparing Asian and Caucasian patients (3, 6). Most GC patients are often diagnosed at the advanced stage and 25–50% of cases will develop metastases during the course of this disease. Surgical resection associated with neoadjuvant and adjuvant therapies remain the primary treatment option for GC patients, but there is an unfavorable prognosis (7). Therefore, this is an unmet need to focus on developing effective diagnostic and therapeutic approaches for GC.

To date, there are two widely accepted histological classification systems for GC: Lauren’s classification that categorizes intestinal and diffuse type (8) and the World Health Organization (WHO) classification dividing GC into adenocarcinoma, papillary adenocarcinoma, tubular adenocarcinoma, mucinous adenocarcinoma, poorly cohesive carcinoma, and mixed carcinoma (9). However, these two classifications are unable to provide specific therapeutic strategies for GC. Recent studies focusing on the molecular classifications of GC have established different molecular subtypes of this disease based on the multi-omics landscape, indicating that GC is a far more complex disease than previously recognized (10).

The Cancer Genome Atlas (TCGA) research group has proposed a molecular classification of four GC subtypes, including Chromosomal instability (CIN), Microsatellite instability (MSI), Genomic stability (GS), and Epstein Barr virus (EBV)-associated, which provide a basis for patient stratification and the development of immunotherapeutic strategies (11). The Asian Cancer Research Group (ACRG) has also defined a similar classification associated with distinct survival: MSI, Microsatellite stable (MSS)/Epithelial-mesenchymal transition (EMT), MSS/TP53^+^, and MSS/TP53^-^ (12). Furthermore, the comprehensive proteogenomic analyses have identified four distinct subtypes of diffuse GC, which are associated with proliferation, immune response, metabolism, and invasion, respectively (13). Further studies are warranted to validate these molecular classifications as well as their respective therapeutic strategies in larger clinical trials in the future.

Recent studies have been performed to systematically investigate the immune characteristics of GC patients, which have adequately demonstrated high prognostic potential and clinical guidance values relative to the conventional clinical characteristics or risk models (14–17). These studies have assessed the immunological characteristics of GC mainly from the perspective of immune cell infiltration. However, there is a lack of investigation regarding the tumor-immune interactions and their prognostic potential in GC. In the present study, we concentrated our efforts on exploring the tumor-immune interaction‐associated molecular characteristics and their prognostic potential. Consequently, we established an immune signature associated with prognosis and demonstrated the stability and reproducibility of this signature in independent datasets by a machine learning approach.

## Methods

### Data Collection and Organization

**Figure 1:**
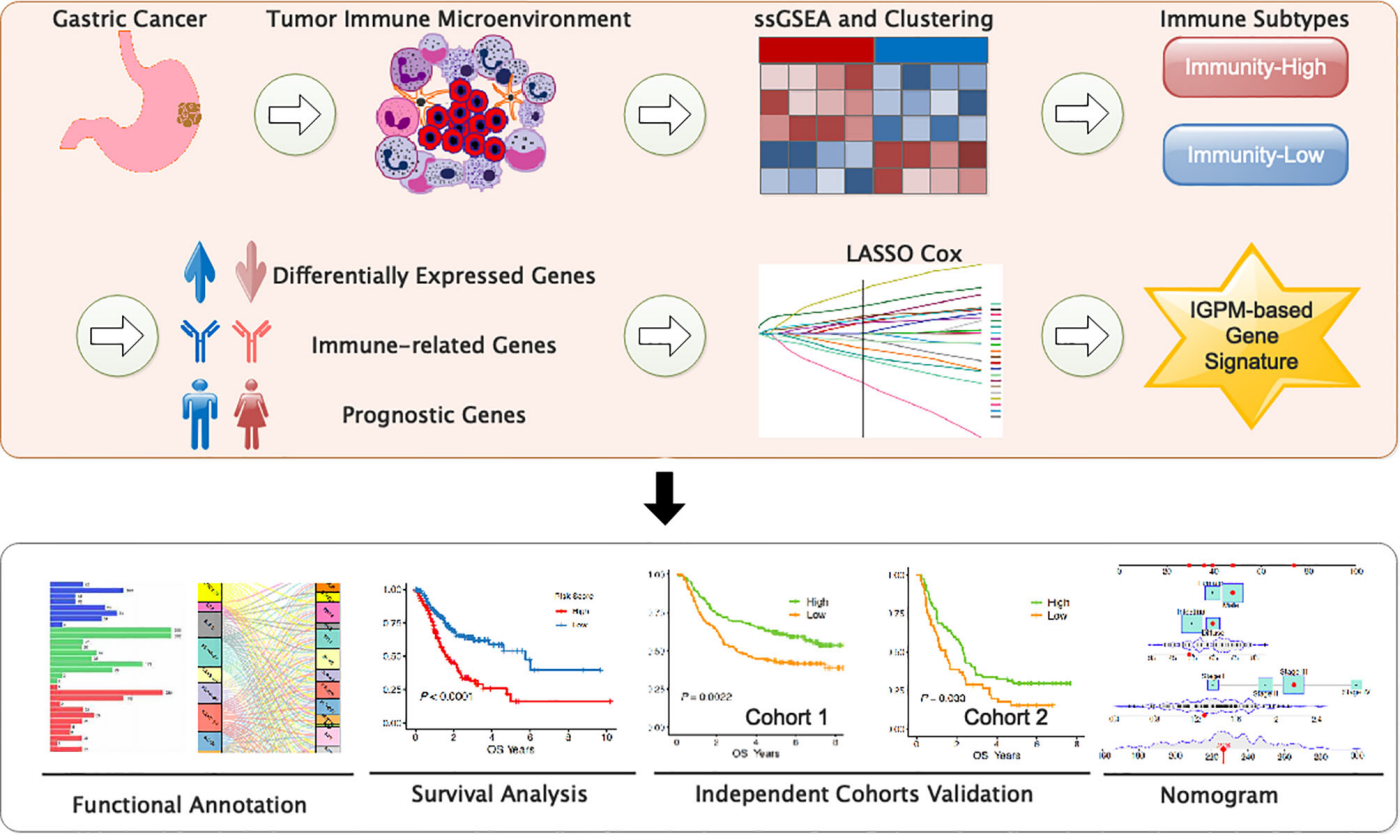
Study flowchart.

We systematically searched the available public transcriptomic cohorts for GC with corresponding clinical information. Normalized gene expression data from RNA-sequencing (RNA-seq; Fragments Per Kilobase Million [FPKM] value) and somatic mutation data and the relevant clinical data of TCGA stomach adenocarcinoma (TCGA-STAD) cohort were downloaded from the University of California Santa Cruz (UCSC) Xena (18). Then, the tumor mutation burden (TMB) per megabase of each sample was calculated as the total number of mutations counted in the exome content. In addition, we retrieved clinical data and normalized gene expression data from the Gene Expression Omnibus (GEO); accession number is GSE62254 and GSE29274. Data were analyzed with R (version 4.0.2) (**Figure 1**).

### Estimation of Immune and Stromal Content, the Proportions of Immune Cell Subsets

We utilized the Estimation of STromal and Immune cells in MAlignant Tumor tissues using Expression data (ESTIMATE) algorithm *via* the R package “estimate” to evaluate the degree of infiltration of tumor cells and different normal cells so as to determine the StromalScore, ImmuneScore, and EstimateScore (19). We used Cell type Identification By Estimating Relative Subsets of RNA Transcripts (CIBERSORT) to calculate the abundance of 22 human infiltrating immune cells for each sample (20).

### Single Sample Gene Sets Enrichment Analysis (ssGSEA) and Hierarchical Clustering Analysis

We employed the ssGSEA algorithm *via* R packages (GSVA, GSEABase, and limma) to comprehensively evaluate the immunological characteristics of each sample included in the study based on 29 immune gene sets, including genes related to different immune cell types, functions, pathways, and checkpoints (21). We transformed each ssGSEA score xi into xi′ by deviation standardization. Then, the subtypes of the GC patients were identified by using hierarchical clustering analysis based on Euclidean distance and Ward’s linkage. The T‐distribution stochastic neighbor embedding (tSNE) algorithm was used *via* the Rtsne package to confirm the accuracy and discrimination of the subtypes of the GC patients (22).

### Differential Expression and Prognosis-Associated Immunity Gene Analyses

We screened the differentially expressed genes (DEGs) (screening conditions: |log2 fold change| > 0.58 and FDR < 0.05) based on the limma package between Immunity High/Low groups and then identified the differentially expressed immunity genes (DEIGs) which were both in the DEGs and Tumor‐associated immune genes downloaded from ImmPort (23, 24). We next performed univariate Cox proportional hazards regression analysis with the survival package in R and considered a gene with P < 0.05 as prognosis‐associated immunity genes (PIGs) from the DEIGs.

### Functional Annotation and Regulation Network Analysis

We conducted a gene-set enrichment analysis of the TCGA-STAD cohort by Gene Set Enrichment Analysis (GSEA, version 4.1.0) to reveal the critical signaling pathways involving the DEGs (25, 26). This analysis identified the Kyoto Encyclopedia of Genes (KEGG) pathways that were upregulated in Immunity_H and Immunity_L, respectively. FDR<0.01 was set as the threshold for screening. Further analysis and visualization were carried out with R (version 4.0.2). Then, we obtained transcription factors related to tumorigenesis and development of GC from the CISTROME project and extracted the differentially expressed transcription factors (DETFs) from the total DEGs and then employed the Pearson’s correlation coefficient analysis to construct the regulatory network of PIGs and DETFs (27, 28). ∣r∣ > 0.3 and FDR < 0.01 were set as the cutoffs for a significant correlation. Protein-protein interaction (PPI) analysis was carried out with STRING (string-db.org/).

### Construction and Validation of Immunity Gene-Associated Prognostic Model

We applied the Cox regression model with LASSO based on the R package “glmnet” to construct an optimal immunity gene‐associated prognostic model (IGPM) for GC by using the PIGs. The Risk score was calculated with the following formula: The $risk\;score=\Sigma_{i=1}^{n}\;(coef_{i}\times Expr_{i})$, where Expr_i_ represents the expression level of gene i and coef_i_ represents the regression coefficient of gene i in the signature. We grouped all patients into low- or high-risk groups according to the median value of IGPM‐based risk signature and performed survival analysis with Kaplan-Meier method. The logrank test was used to compare the difference in the survival status between the high‐ and low‐risk groups. To reflect the prediction ability of the IGPM‐based risk signature, we generated the time-dependent receiver operating characteristic curve (ROC) and calculated the area under the curve (AUC) for 1-year, 3-year, and 5-year overall survival (OS). The Kaplan-Meier, log‐rank, ROC curve, and calibration analyses were all performed and visualized by the “timeROC”, “rms”, “survival”, and “survminer” packages. The relativity between IGPM‐based risk signature and clinical factors, immune cell infiltration, immune checkpoint molecules, and TMB were analyzed using Pearson’s correlation or Spearman correlation with the corrplot package. *P*  < 0.05 were set as the cutoffs for a significant correlation. Finally, we subjected the IGPM‐based risk signature and other clinicopathological characteristics to univariate and multivariate cox regression analyses to confirm the independence of the IGPM‐based risk signature. Then, we used the above factors to establish a nomogram with the R packages “rms”, “nomogramEx”, and “regplot”. Next, ROC and calibration curves analysis were used to determine whether our established nomogram was suitable for clinical use.

## Results

### Immunogenomic Profiling Identifies Two Subtypes of GC

**Figure 2:**
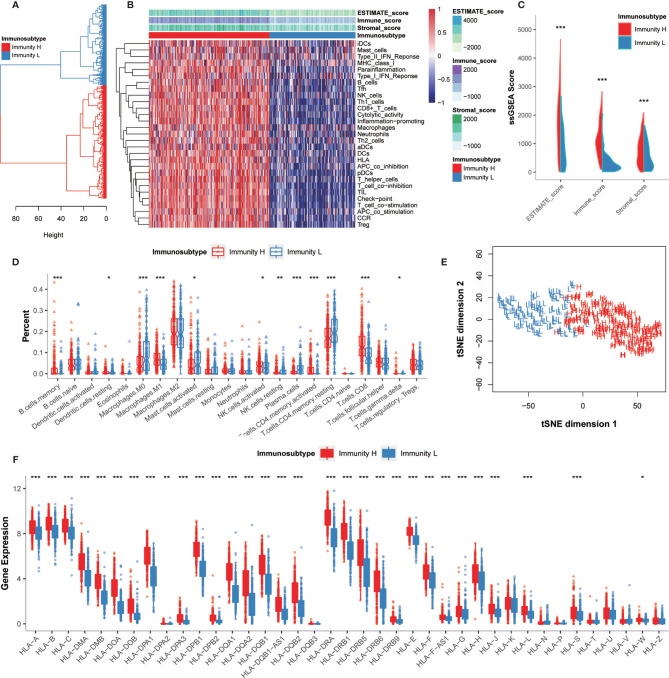
Hierarchical clustering of GC patients discovered two immunophenotypes. **(A)** Hierarchical clustering divided GC patients into Immunity_H and Immunity_L based on the ssGSEA results. **(B)** Landscape of the immune characteristics and tumor microenvironment in the TCGA-STAD cohort. **(C)** Comparison of ESTIMATE_Score, Stromal_score, and Immune_score between two subtypes. **(D)** Comparison of the immune cell infiltration levels between two subtypes. **(E)** Validation of immunophenotype *via* tSNE. **(F)** Comparison of the expression levels of HLA genes between two subtypes. *P < 0.05, **P < 0.01, ***P < 0.001.

To comprehensively evaluate the immunological characteristics in GC, we analyzed 375 tumor samples from the TCGA-STAD cohort using the ssGSEA algorithm along with 29 immune gene sets. Based on the single sample Gene Sets Enrichment Analysis (ssGSEA) scores and hierarchical clustering algorithm, all samples were clearly clustered into two categories: Immunity_H (High) and Immunity_L (Low) (**Figures 2A, B**). According to the results of ESTIMATE, the tumor microenvironment characteristics between these two subtypes were identified. We found that the Immunity_H had higher levels of EstimateScore, ImmuneScore, and StromalScore, whereas Immunity_L had the lower levels of these scores (Wilcox test, *P *< 0.001) (**Figure 2C**).

The degrees of immune cell infiltration were determined using the CIBERSORT algorithm. We found that the infiltration degrees of CD8^+^ T cells, activated memory CD4^+^ T cells, resting memory CD4^+^ T cells, gamma delta T cells, memory B cells, activated NK cells, resting NK cells, M0 macrophages, M1 macrophages, activated mast cells, resting dendritic cells, and plasma cells were significantly different between Immunity_H and Immunity_L groups (**Figure 2D**). We further used the tSNE algorithm to confirm the immune level clustering of the GC patients, and the same classification results were obtained (**Figure 2E**). We also examined the expression of HLA genes in these two subtypes and found that most HLA genes showed significantly higher expression levels in Immunity_H but significantly lower expression levels in Immunity_L (Wilcox test, *P* < 0.05) (**Figure 2F**). Taken together, these results indicated that the immunophenotyping of GC patients was successfully performed.

### Molecular Characteristics and Multidimensional Functional Annotation Describing the Tumor-Immune Interactions and Their Potential Use for Prognostic Prediction

**Figure 3:**
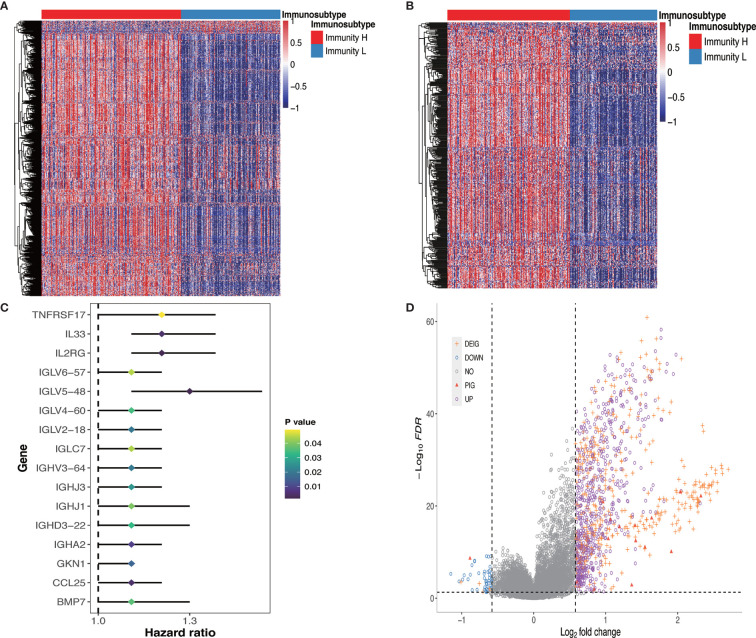
DEGs, DEIGs, and PIGs expression and their prognostic value analysis. **(A)** Landscape of all DEGs between two subtypes. **(B)** Landscape of all DEIGs between two subtypes. **(C)** Forest plot based on univariable Cox proportional hazards regression analysis shows the PIGs and their hazard ratios. **(D)** Volcano plot of all DEGs, DEIGs, and PIGs shows the log_2_ (fold change) and FDR value of each gene. Differentially expressed genes to be exhibited in heatmap graph with |fold change| >1.5 and FDR<0.05. DEG, differentially expressed gene; DEIG, differentially expressed immunity gene; PIG, prognosis-associated immunity gene.

We further investigated the molecular characteristics of tumor-immune interactions and their prognostic potential in GC patients stratified by immunophenotype. After the preliminary screening, a total of 1,086 genes were identified as DEGs. Of these, 1,036 genes were upregulated and 50 genes were downregulated in the Immunity_H group. All DEG expression levels in GC patients were shown in **Figure 3A**. Subsequently, 608 genes were identified as DEIGs based on the ImmPort database, of which 603 genes were upregulated and five genes were downregulated (**Figure 3B**). Finally, 16 PIGs were identified using univariable Cox proportional hazards regression analysis, and the hazard ratio (HR) was calculated (**Figure 3C**). Of these, 15 genes (*IGHJ3*, *IGLV6-57*, *TNFRSF17*, *IGLV2-18*, *IGHD3-22*, *IGHV3-64*, *IL2RG*, *IGHJ1*, *IGLV4-60*, *IGLV5-48*, *IGLC7*, *IGHA2*, *IL33*, *GKN1*, *CCL25*) were upregulated and only 1 gene (*BMP7*) were downregulated in the Immunity_H group. *BMP7*, bone morphogenetic protein 7, can regulate immune cell responses and are immunosuppressive in cancer, which can act as a potential immunotherapeutic target (29). The log2 fold change values in the DEGs, DEIGs, PIGs, and their FDR values in both Immunity_H and Immunity_L groups were shown in **Figure 3D**.

**Figure 4:**
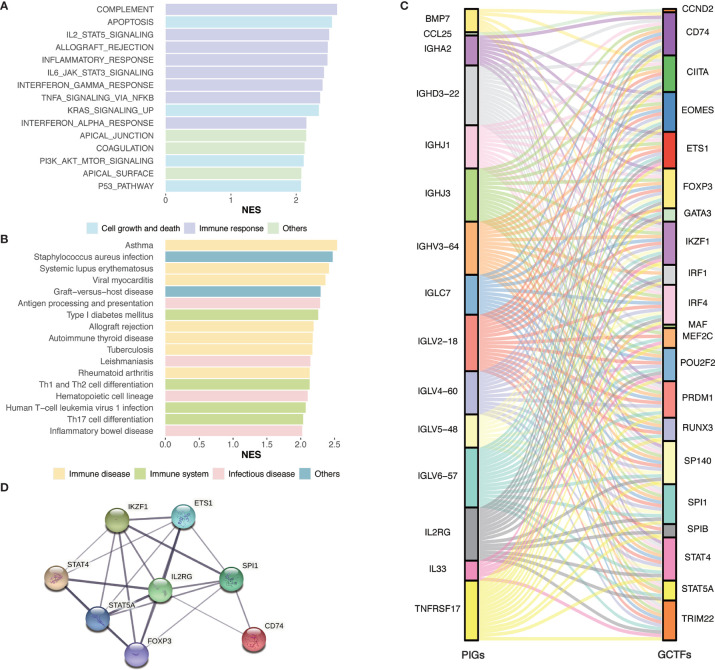
Identification of subtype-specific pathways and networks. **(A)** Hallmark gene sets enrichment analysis of DEGs. **(B)** KEGG enrichment analysis of DEGs. **(C)** Alluvial diagram of the GCTFs and PIGs revealed their regulatory network. **(D)** PPI network between GCTFs and PIGs. Network nodes represent proteins and edges represent protein-protein associations, including both functional and physical protein associations. Line thickness indicates the strength of data support. The thicker line represents the higher confidence. KEGG, Kyoto Encyclopedia of Genes and Genomes; GCTF, gastric cancer transcription factors; PIGs, prognosis‐associated immunity genes.

To gain insight into the global patterns of the effects of immune gene expression on the biological processes in GC, the GSEA was performed to identify the pathways involved in the DEGs. We obtained several biologically sensible themes enriched in Immunity_H, which proved that the DEGs were mainly involved in the immune‐related biological processes or signaling pathways. For the Hallmark gene sets (**Figure 4A** and **Supplementary Table 1.1**), typically, the first group relates to the immune response, including complement, IL2-STAT5 signaling, allograft rejection, inflammatory responses, IL6-JAK-STAT3 signaling, interferon gamma response, TNF-α signaling *via* NF-κB, and interferon alpha response. The second group relates to cell growth and death, including apoptosis, p53 pathway, and transduction pathways such as KRAS signaling and PI3K-Akt signaling.

As for the KEGG pathways (**Figure 4B** and **Supplementary Table 1.2**), the immune-associated pathways were highly active in Immunity_H and included antigen processing and presentation pathways, Th1 and Th2 cell differentiation, Th17 cell differentiation, and hematopoietic cell lineage. Besides, we identified various immune disease-associated pathways that were hyperactivated in Immunity_H, including asthma, systemic lupus erythematosus, graft-versus-host disease, allograft rejection, autoimmune thyroid disease, inflammatory bowel disease, and rheumatoid arthritis. Several pathways related to infectious diseases such as *Staphylococcus aureus* infection, leishmaniasis, tuberculosis, and human T-cell leukemia virus 1 infection were also enriched in Immunity_H.

Considering DEGs used in enrichment analysis were calculated based on 29 immune gene sets, we excluded the genes in the 29 gene sets and reran the enrichment analysis. The results were showed in the **Supplementary Table 2**, which were consistent with the above conclusion (**Figures 4A, B** and **Supplementary Table 1**). In summary, the GSEA result confirmed the elevated immune activity in Immunity_H and showed that the activation of various cancer-associated pathways positively correlated with immune activation.

To elucidate the role of the multidimensional regulatory network of immune molecules in the process of tumorigenesis and development of GC, we first explored the upstream mechanisms of the PIGs. The transcription factors related to the development of GC were identified by combining differential expression analysis and data from the CISTROME database. A total of 23 upregulated transcription factors in the Immunity_H group were identified. Then, the regulatory relationships of the GCTF-PIGs were revealed based on the correlation analysis and all GCTFs were positively correlated with the corresponding PIGs except *BMP7* (**Supplementary Table 3**). The regulatory network of the GCTF-PIGs was shown in **Figure 4C**. To suspect the significant correlations between the GCTF and PIGs, we further performed the PPI analysis and confirmed that there were interactions between them (**Figure 4D**).

### Construction and Validation of the IGPM-Based Risk Signature

**Figure 5:**
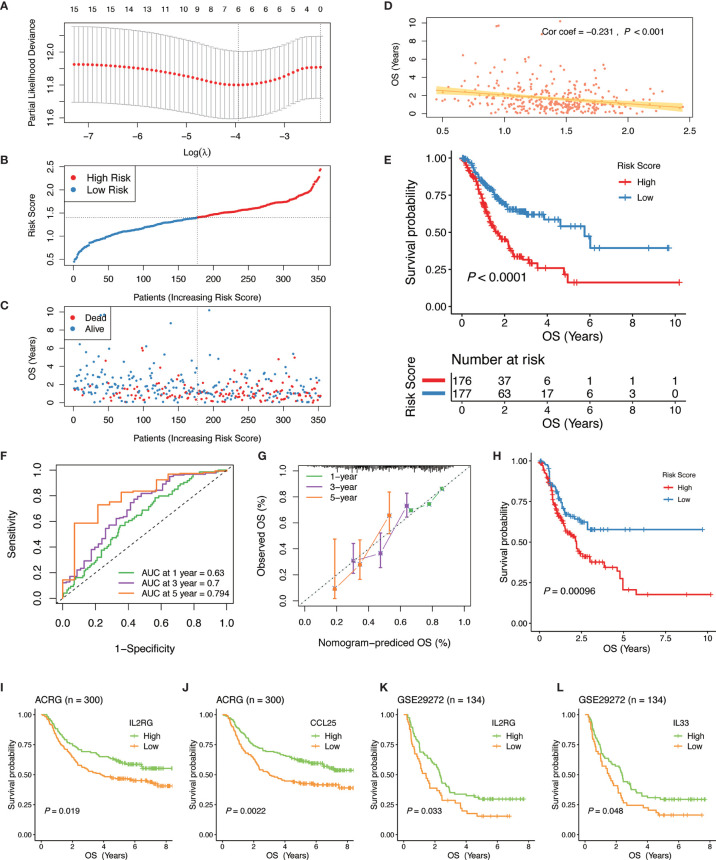
Construction and validation of the IGPM-based risk signature. **(A)** 10-fold cross-validation for tuning parameter selection in the LASSO model. **(B, C)** Distribution of the risk score, survival time, and survival status in the TCGA-STAD cohort. **(D)** Correlation analysis of the risk score and survival time in the TCGA-STAD cohort. **(E)** Kaplan-Meier survival analysis based on the IGPM-based risk signature in the TCGA-STAD cohort. **(F)** The ROC curve and AUC of the predictions for 1, 3, and 5 years of the IGPM-based risk signature for TCGA-STAD cohort. **(G)** The calibration plot of IGPM-based risk signature for TCGA-STAD cohort. **(H)** Kaplan-Meier survival analysis based on the IGPM-based risk signature of the validation cohort that was constructed using the bootstrap method. **(I–L)** Kaplan-Meier survival analysis of the kIGs *IL2RG*, *CCL25*, or *IL33* in the ACRG and GSE29272 cohort. IGPM, immunity gene-associated prognostic model; ROC, curve receiver operating characteristic curve; AUC, area under the curve; kIG, key PIG involved in the construction of the IGPM.

To develop a gene signature based on GC immunophenotype, the PIGs were submitted to LASSO Cox regression analysis for dimension reduction. Consequently, an IGPM‐based risk signature including six genes to predict OS in the TCGA-STAD cohort was constructed. The formula was determined as follows: 0.142 * expression of *IL2RG* + 0.104 * expression of *IGLV5-48* + 0.147 * expression of *BMP7* + 0.110 * expression of *IL33* + 0.010 * expression of *GKN1* + 0.045 * expression of *CCL25* (**Figure 5A**).

We calculated the risk score of each GC patient and divided all patients into the high‐ and low‐risk groups according to a median risk score (**Figure 5B**). Compared with the low‐risk group, the survival time of patients in the high‐risk group was shorter and the incidence of death was higher (**Figure 5C**). The correlation analysis showed that the risk score had a significant negative correlation with the survival time, indicating that the survival times of GC patients gradually decrease with an increasing risk score (**Figure 5D**). The Kaplan-Meier curve was employed for the survival analysis of GC patients and the results showed that the high-risk group was significantly associated with poorer OS, while the low-risk group was associated with better OS (Log-rank test, *P* < 0.0001, **Figure 5E**). To assess the predictive ability and accuracy of the IGPM-based risk signature, we performed a time-dependent ROC curve analysis, and the AUCs of the 1‐, 3‐, and 5‐year predictions were 0.63, 0.7, and 0.794, respectively (**Figure 5F**). The discriminative ability of the IGPM‐based risk signature was measured by determining the calibration plot, which showed that the predicted value of the IGPM‐based risk signature was in a good agreement with the actual value (**Figure 5G**).

We further measured the robustness of the IGPM‐based risk signature by validating its performance in other independent cohorts. We accessed the GEO database recently and did not find a data set that contained both clinical data and all kIGs. To verify the signature more accurately, a bootstrap method was used to obtain the validation cohort with a sample size of 500 from the TCGA-STAD cohort. Survival analysis based on the Kaplan-Meier method showed that there was a significant difference between the high‐risk group and the low‐risk group, suggesting that the IGPM‐based risk signature worked well in the validation cohort (**Figure 5G**). The survival analyses of the kIGs in the ACRG and GSE29272 cohorts were used to validate the predictive ability of the IGPM‐based risk signature once again. The high and low expression groups of *IL2RG*, *CCL25*, or *IL33* had significantly different survival probabilities (**Figure 5I–L**), and the results and trends were consistent with the outcomes of OS prediction for the TCGA-STAD cohort.

### Interrelation of the Risk Score, Clinical Features, Immune Cell Infiltration, Immune Checkpoint Molecules, and TMB

**Figure 6:**
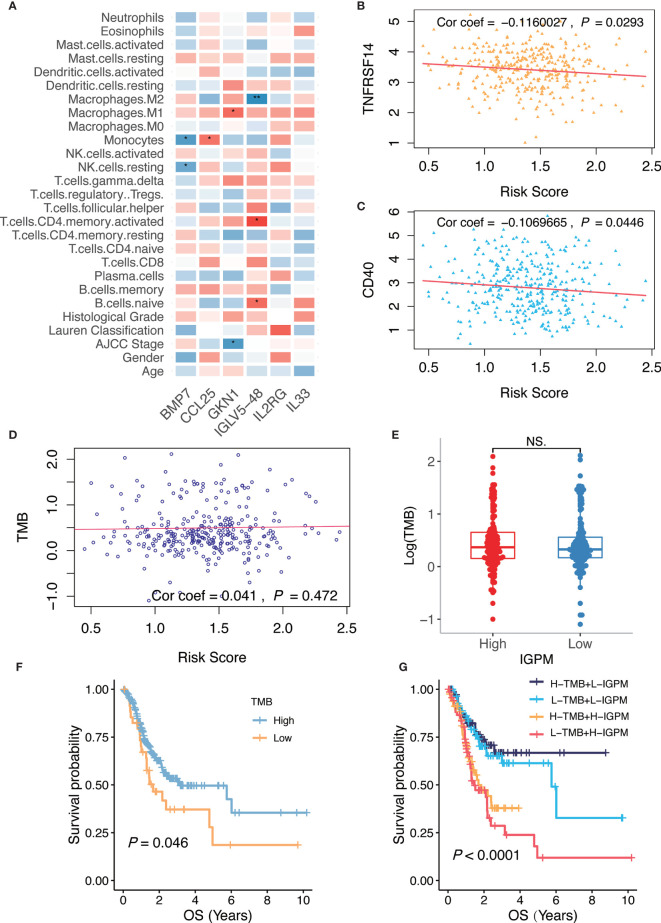
Relationship of the IGPM-based risk signature with kIGs, clinical characteristics, immune microenvironment, and TMB. **(A)** Heatmap showing correlation of the IGPM-based risk signature with kIGs, clinicopathological factors, and immune cell infiltration. The red indicated positive correlation while blue indicated negative correlation. **(B)** Scatter plots depicting correlation between the IGPM-based risk signature and gene expression of *TNFRSF14*. **(C)** Scatter plots depicting correlation between the IGPM-based risk signature and gene expression of *CD40*. **(D)** Scatter plots depicting correlation between the IGPM-based risk signature and TMB. **(E)** Comparison of TMB between IGPM-High and IGPM-low groups. **(F)** Kaplan-Meier survival analysis based on the TMB in the TCGA-STAD cohort. **(G)** Kaplan-Meier survival analysis for four groups stratified by combining the TMB and the IGPM-based risk signature in the TCGA-STAD cohort. *P < 0.05, **P < 0.01. NS, not significant.

We examined the pairwise correlations of the risk score with clinical features, immune cell infiltration, immune checkpoint molecules, and TMB, respectively (**Figure 6A**). Our results showed that *GKN1* had a significant negative correlation with American Joint Committee on Cancer (AJCC) Stage, which indicated that the kIGs might affect the prognosis of GC patients. In terms of the immune microenvironment, *BMP7* had a significant negative correlation with the resting NK cells and monocytes, whereas *CCL25* had a significant positive correlation with monocytes. Besides, *GKNI* had a positive correlation with M1 macrophages. *IGLV5-48* had a positive correlation with naïve B cells and activated CD4 memory T cells, but it showed a significant negative correlation with M2 macrophages.

Next, we aimed to explore whether the risk score could predict immunotherapeutic benefits in GC patients. We selected 14 immune checkpoint-related candidate genes (*BTLA*, *GITR*, *TNFRSF14*, *IDO*, *LAG-3*, *PD-1*, *PD-L1*, *PD-L2*, *CD28*, *CD40*, *CD80*, *CD137*, *CD27*, and *CTLA-4*) to assess their relationships with the risk scores. The results showed that the risk scores were negatively associated with the expression of the critical immune checkpoints (*TNFRSF14* and *CD40*) (**Figures 6B, C**), indicating that the poor prognosis of high IGPM patients might be due to the tumor immunosuppressive microenvironment.

We further found that there was no significant correlation between the risk score and TMB representing nonsynonymous variants (**Figure 6D**). Also, there was no significant difference in TMB between patients with high IGPM and low IGPM (**Figure 6E**). However, we found that high TMB was associated with good OS (Log-rank test, *P* < 0.05, **Figure 6F**). Considering that the TMB predictive value was not very high in the TCGA-STAD cohort, we explored whether the combination of IGPM and TMB could be a more powerful predictive biomarker for the prognosis. We integrated IGPM and TMB to stratify all the samples into high TMB/low IGPM, low TMB/low IGPM, high TMB/high IGPM, and low TMB/high IGPM groups. As shown in **Figure 6G**, significant differences were found among all groups (Log-rank test,  *P* < 0.0001), and patients in the high TMB/low IGPM group exhibited the best OS. These results together strongly demonstrated that the risk score was positively correlated with tumor malignancy.

### Establishment and Validation of a Nomogram Combined With Clinical Characteristics

As the risk score was significantly correlated with high malignancy, we examined whether the IGPM‐based risk signature could serve as an independent prognostic factor for GC patients through univariate and multivariate Cox regression analysis. The IGPM‐based risk signature, together with age, gender, AJCC stage, and Lauren classification were enrolled as covariates to perform the analysis. The results demonstrated that the p value of the IGPM‐based risk signature was less than 0.0001, confirming that the IGPM‐based risk signature could be utilized to predict the prognosis of GC patients (**Table 1**).

**Table 1:** Univariable and multivariable Cox analyses for clinical characteristics and IGPM in the TCGA-STAD cohort.

Variables	Univariable Cox analysis		Multivariable Cox analysis	
	**Hazard ratio (95% CI)**	**p value**	**Hazard ratio (95% CI)**	**p value**
**Age**	1 (1–1)	**0.01**	1.04 (1.01–1.06)	**0.002**
**Gender**				
Female	1			
Male	1.3 (0.91–1.8)	0.15	1.2 (0.76–1.89)	0.431
**AJCC Stage**				
Stage I	1			
Stage II	1.55 (0.78–3.08)	**3e-04**	1.98 (0.77–5.08)	0.157
Stage III	2.39 (1.26–4.52)	**5e-04**	2.88 (1.23–6.75)	**0.015**
Stage IV	3.83 (1.85–7.90)	**3e-04**	8.86 (3.44–22.79)	**6.07e-06**
**Lauren Classification**				
Diffuse	1			
Intestinal	0.84 (0.55–1.3)	0.43	0.68 (0.43–1.07)	0.093
**IGPM**				
High	1			
Low	0.43 (0.31–0.61)	**1.6e-06**	0.39 (0.25–0.61)	**3.13e-05**

The bold values mean they are statistically significant.

**Figure 7:**
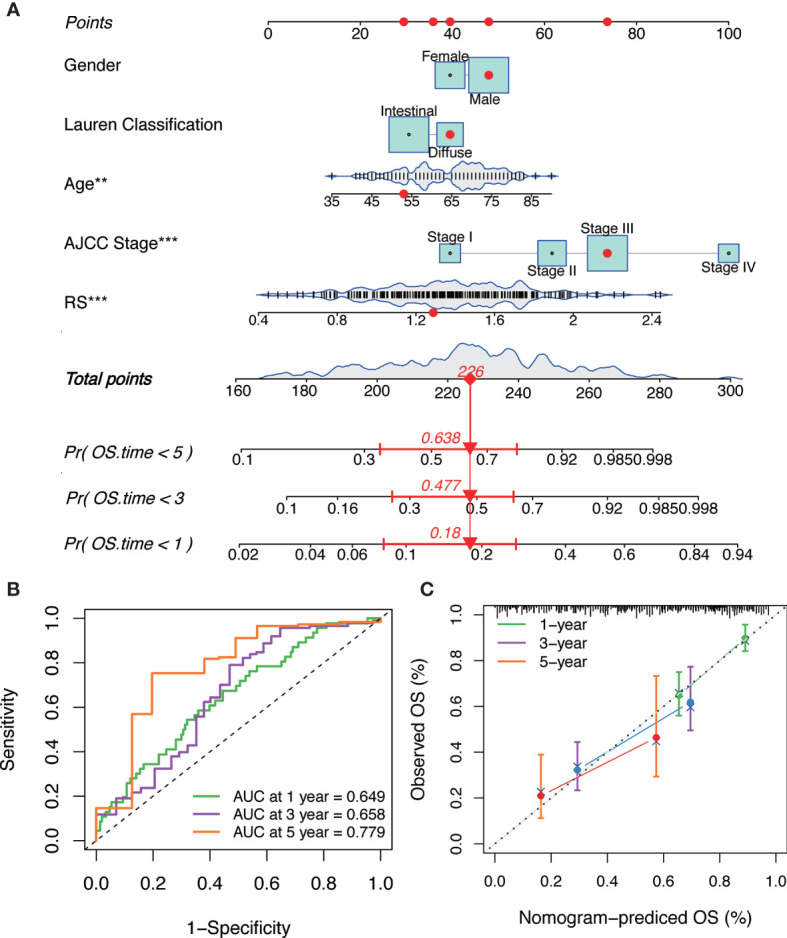
The construction and verification of the nomogram. **(A)** Nomogram constructed in conjunction with the IGPM-based risk signature and clinical characterization. **(B)** The ROC curve and AUC of the predictions for 1, 3, and 5 years of the nomogram for TCGA-STAD cohort. **(C)** The calibration plot of the nomogram for TCGA-STAD cohort.

Combining the above factors, we constructed a nomogram to broaden the clinical application and usability of the IGPM‐based risk signature (**Figure 7A**). Every patient was assigned with a total point value by adding the point for each prognostic parameter. Higher total points corresponded to a worse clinical outcome of patients. The ROC curve indicated that the nomogram had good predictive ability and accuracy for survival status (**Figure 7B**). Furthermore, the calibration plot showed that the nomogram had similar performance to that of an ideal model (**Figure 7C**).

## Discussion

In recent years, GC classification on the basis of multi-omics profiling has been extensively investigated, and these efforts might lay the foundation for developing new biomarkers and drug targets for GC (30, 31). It has also been demonstrated that the stratification of GC patients based on immunological characteristics may improve the survival rates of patients (32); however, the tumor-immune interactions were not considered sufficiently in these studies. In the present study, we identified two immune-related GC subtypes, Immunity_H and Immunity_L using immunogenomic profiling, and characterized the subtype-specific molecular features, including genes, pathways, and networks.

Immunity_H had stronger immune cell infiltration and higher expressed HLA genes, indicating stronger immunogenicity compared to Immunity_L. The Immunity_H subtype was not only enriched in immune-related signatures but also many cancer-associated pathways, including apoptosis, p53 signaling, PI3K-Akt signaling, and KRAS signaling. These results are consistent with previous reports showing that diverse immune signatures are positively correlated with the JAK-STAT3 and PI3K-Akt signaling pathways (33, 34). Importantly, our results revealed the potential associations between pathway activities and immune activities in GC.

Based on the expression of six immune genes, we developed the IGPM-based risk signature, a novel prognostic tool for predicting survival in GC, and demonstrated its predictive value of prognosis in three independent datasets by a machine learning approach. The results showed a clear separation of OS curves between patients with high- and low-risk scores. The predictive value of the IGPM-based risk signature was also consistent with that of clinical variables and immune cell infiltrates.

Among six genes used to construct the IGPM-based risk signature, five of them have been reported to contribute to carcinogenesis and may be promising therapeutic targets. *IL2RG* (interleukin-2 receptor subunit gamma), a gene encoding the IL2 gamma receptor (Il2Rγ) is overexpressed in pancreatic intraepithelial neoplasia and induces pancreatic cancer growth by activating the JAK/STAT pathway (35). *IL33* (interleukin 33) induces the phosphorylation of c-Jun N terminal kinase (JNK), recruits macrophages into the cancer microenvironment, and promotes the carcinogenesis of colon cancer (36). *CCL25* (chemotactic cytokines 25) can promote the proliferation by activating the AKT signaling and intratumoral delivery of *CCL25* may enhance immunotherapy (37). *BMP7*, a member of the TGFB superfamily, inhibits the MAPK14 signaling pathway and induces resistance to anti-PD1 therapy (29). Gastrokine 1 (*GKN1*) has been found to suppress GC cell growth by activating the p16/Rb pathway (38). *IGLV5-48* (Immunoglobulin Lambda Variable 5-48) is a protein coding gene; however, its function and molecular mechanism are not clear. This prompted us to further investigate the molecular mechanisms of these genes in GC in future studies.

Of note, we observed two immune checkpoint genes *TNFRSF14* and *CD40* had a significant negative correlation with the IGPM-based risk signature, which is consistent with previous studies. It has been reported that *TNFRSF14* loss results in microenvironmental changes that support lymphoma growth and the expression of *TNFRSF14* plays a role in the pathobiology and prognosis in follicular lymphoma (39, 40). Moreover, CD40 activation plays a critical role in generating T cell immunity, especially in combination with chemotherapy, checkpoint inhibitory antibodies, and other immune modulators (41).

In this study, we concluded that GC patients with higher TMB had a better prognosis compared with those with lower TMB. Similar results have been obtained in other cancer types, emphasizing that TMB could be an independent biomarker to guide more effective immunotherapy strategies and bring out improved prognosis (42, 43). However, we did not observe a significant correlation between the IGPM-based risk signature and TMB. The stratified survival analysis combining TMB and the IGPM-based risk signature revealed that the TMB status did not interfere with the prognostic value of the IGPM-based risk signature. These findings together indicated that the IGPM-based risk signature might help facilitate the measurement of the response to immunotherapy.

## Conclusion

In summary, we have elucidated the comprehensive landscape of tumor-immune interactions in GC and established the IGPM-based risk signature to predict the prognosis of GC patients with the help of several computational algorithms. As all patients in this study were selected retrospectively, the six-gene signature should be further validated in prospective cohorts of high-quality clinical samples to reduce the potential bias derived from unbalanced clinicopathological features with treatment heterogeneity. Further investigations on detailed molecular mechanisms are needed to facilitate the precision immunotherapy, as well as the accurate and reliable applications in the clinic.

## Data Availability Statement

All data used in this work can be acquired from the UCSC Xena (https://tcga.xenahubs.net) and the GEO (https://www.ncbi.nlm.nih.gov/geo/). The accession number(s) can be found in the article/**Supplementary Material**.

## Author Contributions

XG led the bioinformatic and biostatistical data analysis. XG and Z-YX collected the literature, wrote the manuscript, and made the figures. RC and J-JQ edited and made significant revisions to the manuscript. J-JQ and X-DC contributed to the study design and project supervision. All authors contributed to the article and approved the submitted version.

## Funding

This study was supported by Natural Science Foundation of Zhejiang Province (LY18H290006), National Natural Science Foundation of China (81903842, 81973634), and Program of Zhejiang Provincial TCM Sci-tech Plan (2018ZY006, 2020ZZ005).

## Conflict of Interest

The authors declare that the research was conducted in the absence of any commercial or financial relationships that could be construed as a potential conflict of interest.
