# Supplementary material for: Identification of an Immune Gene-Associated Prognostic Signature and Its Association With a Poor Prognosis in Gastric Cancer Patients
Source: Front Oncol. 2021 Feb 8;10:629909. doi: 10.3389/fonc.2020.629909 (PMC7898907; doi:10.3389/fonc.2020.629909)
Supplement: Supplementary file 1 [file Table_1.docx]

**Supplementary Table 1.1 Hallmark genesets enriched in Immunity_H group**

| NAME | NES | FDR |
| --- | --- | --- |
| COMPLEMENT | 2.6278434 | 0 |
| APOPTOSIS | 2.5505648 | 8.52E-04 |
| IL2_STAT5_SIGNALING | 2.5061948 | 5.68E-04 |
| ALLOGRAFT_REJECTION | 2.4840877 | 4.26E-04 |
| INFLAMMATORY_RESPONSE | 2.4794643 | 3.41E-04 |
| IL6_JAK_STAT3_SIGNALING | 2.426317 | 4.84E-04 |
| INTERFERON_GAMMA_RESPONSE | 2.4048264 | 5.55E-04 |
| TNFA_SIGNALING_VIA_NFKB | 2.3670936 | 6.15E-04 |
| KRAS_SIGNALING_UP | 2.3474479 | 5.46E-04 |
| INTERFERON_ALPHA_RESPONSE | 2.157268 | 0.0045747 |
| APICAL_JUNCTION | 2.1533017 | 0.00432509 |
| COAGULATION | 2.1287653 | 0.00541445 |
| PI3K_AKT_MTOR_SIGNALING | 2.115677 | 0.00573054 |
| APICAL_SURFACE | 2.077842 | 0.00727967 |
| P53_PATHWAY | 2.0746427 | 0.00686641 |

**Supplementary Table 1.2 KEGG genesets enriched in Immunity_H group**

| NAME | NES | FDR |
| --- | --- | --- |
| Asthma | 2.5372104 | 6.96E-05 |
| Staphylococcus aureus infection | 2.47150985 | 5.18E-05 |
| Systemic lupus erythematosus | 2.41734481 | 0.0001471 |
| Viral myocarditis | 2.36497257 | 0.00027253 |
| Graft-versus-host disease | 2.29344044 | 0.00065852 |
| Antigen processing and presentation | 2.28588604 | 0.00065852 |
| Type I diabetes mellitus | 2.25802742 | 0.00065852 |
| Allograft rejection | 2.18921982 | 0.00091963 |
| Autoimmune thyroid disease | 2.17651217 | 0.00091963 |
| Tuberculosis | 2.17193508 | 0.00091963 |
| Leishmaniasis | 2.14146112 | 0.0010858 |
| Rheumatoid arthritis | 2.13132868 | 0.0010858 |
| Th1 and Th2 cell differentiation | 2.13078059 | 0.0010858 |
| Hematopoietic cell lineage | 2.10204806 | 0.00094454 |
| Human T-cell leukemia virus 1 infection | 2.0735604 | 0.0010858 |
| Th17 cell differentiation | 2.03535302 | 0.00529677 |
| Inflammatory bowel disease | 2.0207008 | 0.00407877 |
