# Supplementary material for: Identification of an Immune Gene-Associated Prognostic Signature and Its Association With a Poor Prognosis in Gastric Cancer Patients
Source: Front Oncol. 2021 Feb 8;10:629909. doi: 10.3389/fonc.2020.629909 (PMC7898907; doi:10.3389/fonc.2020.629909)
Supplement: Supplementary file 2 [file Table_2.docx]

**Supplementary Table 2.1 Hallmark genesets enriched in Immunity_H group**

| NAME | NES | FDR |
| --- | --- | --- |
| COMPLEMENT | 1.1254257 | 1 |
| ALLOGRAFT_REJECTION | 1.0540557 | 1 |
| KRAS_SIGNALING_UP | 1.0490766 | 1 |
| INFLAMMATORY_RESPONSE | 0.8387265 | 1 |
| INTERFERON_GAMMA_RESPONSE | 0.7939673 | 1 |
| INTERFERON_ALPHA_RESPONSE | 0.6923818 | 1 |
| COAGULATION | 0.68405145 | 0.9609216 |
| IL2_STAT5_SIGNALING | 0.5437425 | 0.9421065 |

**Supplementary Table 2.2 Hallmark genesets enriched in Immunity_L group**

| NAME | NES | FDR |
| --- | --- | --- |
| EPITHELIAL_MESENCHYMAL_TRANSITION | -3.163944 | 0 |
| MYOGENESIS | -1.3887962 | 0.22010715 |
| APOPTOSIS | -1.2832338 | 0.2393307 |
| APICAL_JUNCTION | -1.2399623 | 0.22003667 |
| TNFA_SIGNALING_VIA_NFKB | -0.9184215 | 0.56449515 |

**Supplementary Table 2.3 KEGG genesets enriched in Immunity_H group**

| NAME | NES | FDR |
| --- | --- | --- |
| COMPLEMENT_AND_COAGULATION_CASCADES | 1.6123534 | 0.1258667 |
| LEUKOCYTE_TRANSENDOTHELIAL_MIGRATION | 0.96621907 | 1 |
| CHEMOKINE_SIGNALING_PATHWAY | 0.8544891 | 0.8744433 |
| REGULATION_OF_ACTIN_CYTOSKELETON | 0.7108253 | 0.8099313 |

**Supplementary Table 2.4 KEGG genesets enriched in Immunity_L group**

| NAME | NES | FDR |
| --- | --- | --- |
| FOCAL_ADHESION | -1.3964092 | 0.16833666 |
| PATHWAYS_IN_CANCER | -0.8343358 | 0.6783567 |
