# Supplementary material for: Identification of an Immune Gene-Associated Prognostic Signature and Its Association With a Poor Prognosis in Gastric Cancer Patients
Source: Front Oncol. 2021 Feb 8;10:629909. doi: 10.3389/fonc.2020.629909 (PMC7898907; doi:10.3389/fonc.2020.629909)
Supplement: Supplementary file 3 [file Table_3.docx]

**Supplementary Table 3. Regulations between PIGs and GCTFs**

| PIGs | GCTFs | Coefficient | P-value | FDR |
| --- | --- | --- | --- | --- |
| BMP7 | CD74 | -0.40553 | 2.8151E-16 | 1.068E-15 |
| BMP7 | CIITA | -0.3424528 | 9.32E-12 | 2.2865E-11 |
| BMP7 | IRF1 | -0.3188694 | 2.6157E-10 | 5.564E-10 |
| BMP7 | SP140 | -0.3037994 | 1.9022E-09 | 3.7634E-09 |
| BMP7 | SPI1 | -0.3463589 | 5.2172E-12 | 1.3241E-11 |
| BMP7 | STAT4 | -0.3027512 | 2.1747E-09 | 4.2795E-09 |
| BMP7 | TRIM22 | -0.3400261 | 1.3311E-11 | 3.1807E-11 |
| CCL25 | CCND2 | 0.34962687 | 3.1909E-12 | 8.2695E-12 |
| IGHA2 | CD74 | 0.34859784 | 3.7275E-12 | 9.5925E-12 |
| IGHA2 | EOMES | 0.30777739 | 1.139E-09 | 2.303E-09 |
| IGHA2 | ETS1 | 0.31220012 | 6.3815E-10 | 1.3119E-09 |
| IGHA2 | FOXP3 | 0.38174995 | 1.8607E-14 | 5.6833E-14 |
| IGHA2 | IKZF1 | 0.45185482 | 2.878E-20 | 1.8581E-19 |
| IGHA2 | IRF4 | 0.60009916 | 4.7102E-38 | 1.7334E-36 |
| IGHA2 | PRDM1 | 0.37879228 | 3.0615E-14 | 9.1597E-14 |
| IGHA2 | SP140 | 0.41793342 | 2.758E-17 | 1.2377E-16 |
| IGHA2 | STAT4 | 0.33822635 | 1.7303E-11 | 4.0817E-11 |
| IGHD3-22 | CD74 | 0.46123302 | 3.751E-21 | 2.5563E-20 |
| IGHD3-22 | CIITA | 0.46229374 | 2.9671E-21 | 2.0602E-20 |
| IGHD3-22 | EOMES | 0.41371475 | 6.144E-17 | 2.5122E-16 |
| IGHD3-22 | ETS1 | 0.40051197 | 7.0125E-16 | 2.4345E-15 |
| IGHD3-22 | FOXP3 | 0.52678016 | 3.6708E-28 | 4.5028E-27 |
| IGHD3-22 | IKZF1 | 0.59150981 | 9.2155E-37 | 3.083E-35 |
| IGHD3-22 | IRF1 | 0.30209754 | 2.3633E-09 | 4.6016E-09 |
| IGHD3-22 | IRF4 | 0.69336734 | 4.898E-55 | 6.0082E-53 |
| IGHD3-22 | MEF2C | 0.35358303 | 1.7462E-12 | 4.5901E-12 |
| IGHD3-22 | POU2F2 | 0.45070345 | 3.6802E-20 | 2.335E-19 |
| IGHD3-22 | PRDM1 | 0.33529232 | 2.6441E-11 | 6.0814E-11 |
| IGHD3-22 | RUNX3 | 0.40540029 | 2.8828E-16 | 1.0716E-15 |
| IGHD3-22 | SP140 | 0.58979549 | 1.6507E-36 | 5.0622E-35 |
| IGHD3-22 | SPI1 | 0.46505269 | 1.6063E-21 | 1.1368E-20 |
| IGHD3-22 | SPIB | 0.40499031 | 3.1077E-16 | 1.1436E-15 |
| IGHD3-22 | STAT4 | 0.46926374 | 6.2282E-22 | 4.6775E-21 |
| IGHD3-22 | STAT5A | 0.39738165 | 1.2296E-15 | 4.1514E-15 |
| IGHD3-22 | TRIM22 | 0.42038964 | 1.721E-17 | 8.1198E-17 |
| IGHJ1 | CD74 | 0.41951773 | 2.0356E-17 | 9.4821E-17 |
| IGHJ1 | CIITA | 0.3365949 | 2.1916E-11 | 5.1045E-11 |
| IGHJ1 | EOMES | 0.35518312 | 1.3651E-12 | 3.6669E-12 |
| IGHJ1 | ETS1 | 0.3478117 | 4.1959E-12 | 1.0723E-11 |
| IGHJ1 | FOXP3 | 0.47686277 | 1.0897E-22 | 8.7175E-22 |
| IGHJ1 | IKZF1 | 0.49472836 | 1.5177E-24 | 1.4321E-23 |
| IGHJ1 | IRF4 | 0.62498691 | 5.0268E-42 | 2.0554E-40 |
| IGHJ1 | PRDM1 | 0.35686284 | 1.0526E-12 | 2.8482E-12 |
| IGHJ1 | SP140 | 0.44827466 | 6.1629E-20 | 3.718E-19 |
| IGHJ1 | SPI1 | 0.40666159 | 2.2865E-16 | 8.765E-16 |
| IGHJ1 | STAT4 | 0.42194966 | 1.273E-17 | 6.1642E-17 |
| IGHJ1 | STAT5A | 0.30215066 | 2.3474E-09 | 4.595E-09 |
| IGHJ1 | TRIM22 | 0.31603531 | 3.8314E-10 | 7.9659E-10 |
| IGHJ3 | CD74 | 0.4898225 | 5.0324E-24 | 4.6298E-23 |
| IGHJ3 | CIITA | 0.394885 | 1.9162E-15 | 6.3529E-15 |
| IGHJ3 | EOMES | 0.41485611 | 4.9525E-17 | 2.0948E-16 |
| IGHJ3 | ETS1 | 0.41475729 | 5.0459E-17 | 2.1101E-16 |
| IGHJ3 | FOXP3 | 0.54205137 | 5.0324E-30 | 8.4178E-29 |
| IGHJ3 | IKZF1 | 0.57410082 | 2.9293E-34 | 7.7E-33 |
| IGHJ3 | IRF4 | 0.71296113 | 1.8259E-59 | 3.3596E-57 |
| IGHJ3 | MEF2C | 0.35388314 | 1.6676E-12 | 4.415E-12 |
| IGHJ3 | POU2F2 | 0.32988086 | 5.7128E-11 | 1.2898E-10 |
| IGHJ3 | PRDM1 | 0.40846789 | 1.6379E-16 | 6.4813E-16 |
| IGHJ3 | RUNX3 | 0.38977917 | 4.6931E-15 | 1.5018E-14 |
| IGHJ3 | SP140 | 0.52721108 | 3.2619E-28 | 4.1393E-27 |
| IGHJ3 | SPI1 | 0.46765177 | 8.9648E-22 | 6.5981E-21 |
| IGHJ3 | STAT4 | 0.48683719 | 1.0339E-23 | 9.0589E-23 |
| IGHJ3 | STAT5A | 0.32673742 | 8.8756E-11 | 1.9676E-10 |
| IGHJ3 | TRIM22 | 0.39916648 | 8.9335E-16 | 3.0725E-15 |
| IGHV3-64 | CD74 | 0.44257219 | 2.0348E-19 | 1.152E-18 |
| IGHV3-64 | CIITA | 0.40827689 | 1.6969E-16 | 6.6432E-16 |
| IGHV3-64 | EOMES | 0.39166204 | 3.379E-15 | 1.0908E-14 |
| IGHV3-64 | ETS1 | 0.40333771 | 4.2023E-16 | 1.5161E-15 |
| IGHV3-64 | FOXP3 | 0.50499603 | 1.1589E-25 | 1.2543E-24 |
| IGHV3-64 | IKZF1 | 0.53427458 | 4.5956E-29 | 6.5045E-28 |
| IGHV3-64 | IRF1 | 0.34559113 | 5.8512E-12 | 1.4648E-11 |
| IGHV3-64 | IRF4 | 0.66503751 | 3.154E-49 | 1.9345E-47 |
| IGHV3-64 | POU2F2 | 0.32432911 | 1.2397E-10 | 2.7318E-10 |
| IGHV3-64 | PRDM1 | 0.43411849 | 1.1475E-18 | 6.0325E-18 |
| IGHV3-64 | RUNX3 | 0.34607429 | 5.444E-12 | 1.3722E-11 |
| IGHV3-64 | SP140 | 0.48239748 | 2.9777E-23 | 2.4351E-22 |
| IGHV3-64 | SPI1 | 0.39433449 | 2.1121E-15 | 6.8784E-15 |
| IGHV3-64 | STAT4 | 0.43397286 | 1.1817E-18 | 6.1249E-18 |
| IGHV3-64 | STAT5A | 0.34350319 | 7.98E-12 | 1.9709E-11 |
| IGHV3-64 | TRIM22 | 0.39676982 | 1.3713E-15 | 4.5877E-15 |
| IGLC7 | CD74 | 0.35149617 | 2.4025E-12 | 6.2704E-12 |
| IGLC7 | EOMES | 0.34067934 | 1.2097E-11 | 2.9287E-11 |
| IGLC7 | FOXP3 | 0.4150987 | 4.7301E-17 | 2.0241E-16 |
| IGLC7 | IKZF1 | 0.44334707 | 1.7322E-19 | 9.9601E-19 |
| IGLC7 | IRF4 | 0.57088737 | 8.1748E-34 | 2.0056E-32 |
| IGLC7 | POU2F2 | 0.37255334 | 8.6094E-14 | 2.5145E-13 |
| IGLC7 | PRDM1 | 0.38689846 | 7.7263E-15 | 2.4096E-14 |
| IGLC7 | RUNX3 | 0.32728541 | 8.2223E-11 | 1.845E-10 |
| IGLC7 | SP140 | 0.43996393 | 3.4877E-19 | 1.8875E-18 |
| IGLC7 | SPI1 | 0.3601518 | 6.2988E-13 | 1.7428E-12 |
| IGLC7 | STAT4 | 0.3897144 | 4.7462E-15 | 1.5057E-14 |
| IGLC7 | TRIM22 | 0.34072832 | 1.201E-11 | 2.927E-11 |
| IGLV2-18 | CD74 | 0.45035839 | 3.9609E-20 | 2.4705E-19 |
| IGLV2-18 | CIITA | 0.35410608 | 1.6114E-12 | 4.2972E-12 |
| IGLV2-18 | EOMES | 0.45022214 | 4.0774E-20 | 2.5008E-19 |
| IGLV2-18 | ETS1 | 0.37589071 | 4.9656E-14 | 1.4619E-13 |
| IGLV2-18 | FOXP3 | 0.50860258 | 4.5983E-26 | 5.288E-25 |
| IGLV2-18 | GATA3 | 0.30891134 | 9.8266E-10 | 1.9979E-09 |
| IGLV2-18 | IKZF1 | 0.53456465 | 4.236E-29 | 6.2354E-28 |
| IGLV2-18 | IRF4 | 0.65862863 | 5.3177E-48 | 2.7956E-46 |
| IGLV2-18 | MEF2C | 0.31799713 | 2.943E-10 | 6.1888E-10 |
| IGLV2-18 | POU2F2 | 0.37246992 | 8.7279E-14 | 2.529E-13 |
| IGLV2-18 | PRDM1 | 0.40093681 | 6.4949E-16 | 2.2763E-15 |
| IGLV2-18 | RUNX3 | 0.33865831 | 1.625E-11 | 3.858E-11 |
| IGLV2-18 | SP140 | 0.55083819 | 3.8541E-31 | 7.4649E-30 |
| IGLV2-18 | SPI1 | 0.41413337 | 5.6774E-17 | 2.3475E-16 |
| IGLV2-18 | SPIB | 0.32316293 | 1.4559E-10 | 3.1516E-10 |
| IGLV2-18 | STAT4 | 0.46087931 | 4.0553E-21 | 2.7134E-20 |
| IGLV2-18 | TRIM22 | 0.39820804 | 1.0608E-15 | 3.6146E-15 |
| IGLV4-60 | CD74 | 0.40307759 | 4.406E-16 | 1.5742E-15 |
| IGLV4-60 | CIITA | 0.33018193 | 5.4753E-11 | 1.2438E-10 |
| IGLV4-60 | EOMES | 0.38172467 | 1.8687E-14 | 5.6833E-14 |
| IGLV4-60 | ETS1 | 0.31361534 | 5.2909E-10 | 1.0939E-09 |
| IGLV4-60 | FOXP3 | 0.41854828 | 2.4518E-17 | 1.1139E-16 |
| IGLV4-60 | IKZF1 | 0.47312567 | 2.5821E-22 | 2.0217E-21 |
| IGLV4-60 | IRF4 | 0.55568165 | 9.0491E-32 | 2.0813E-30 |
| IGLV4-60 | POU2F2 | 0.30200058 | 2.3926E-09 | 4.6342E-09 |
| IGLV4-60 | PRDM1 | 0.35916898 | 7.3479E-13 | 2.0179E-12 |
| IGLV4-60 | SP140 | 0.41053717 | 1.1149E-16 | 4.4597E-16 |
| IGLV4-60 | SPI1 | 0.36031303 | 6.1413E-13 | 1.7121E-12 |
| IGLV4-60 | STAT4 | 0.37108797 | 1.0941E-13 | 3.1454E-13 |
| IGLV4-60 | TRIM22 | 0.35814114 | 8.6277E-13 | 2.3518E-12 |
| IGLV5-48 | CD74 | 0.40389453 | 3.7968E-16 | 1.3834E-15 |
| IGLV5-48 | CIITA | 0.30920891 | 9.4523E-10 | 1.9325E-09 |
| IGLV5-48 | EOMES | 0.31875293 | 2.6573E-10 | 5.62E-10 |
| IGLV5-48 | FOXP3 | 0.4054687 | 2.8469E-16 | 1.069E-15 |
| IGLV5-48 | IKZF1 | 0.4189088 | 2.2881E-17 | 1.0525E-16 |
| IGLV5-48 | IRF4 | 0.54345427 | 3.356E-30 | 6.175E-29 |
| IGLV5-48 | PRDM1 | 0.32225039 | 1.6502E-10 | 3.5514E-10 |
| IGLV5-48 | SP140 | 0.42485945 | 7.2239E-18 | 3.5924E-17 |
| IGLV5-48 | SPI1 | 0.38036508 | 2.3508E-14 | 7.0909E-14 |
| IGLV5-48 | STAT4 | 0.32036097 | 2.1362E-10 | 4.5705E-10 |
| IGLV6-57 | CD74 | 0.50135042 | 2.9176E-25 | 2.9824E-24 |
| IGLV6-57 | CIITA | 0.38769925 | 6.7297E-15 | 2.1167E-14 |
| IGLV6-57 | EOMES | 0.41560153 | 4.3001E-17 | 1.8617E-16 |
| IGLV6-57 | ETS1 | 0.40783585 | 1.8412E-16 | 7.1321E-16 |
| IGLV6-57 | FOXP3 | 0.5325069 | 7.5374E-29 | 1.0273E-27 |
| IGLV6-57 | GATA3 | 0.30591481 | 1.4495E-09 | 2.9149E-09 |
| IGLV6-57 | IKZF1 | 0.55299711 | 2.0262E-31 | 4.1425E-30 |
| IGLV6-57 | IRF1 | 0.3271345 | 8.3974E-11 | 1.8729E-10 |
| IGLV6-57 | IRF4 | 0.68832854 | 5.9148E-54 | 5.4417E-52 |
| IGLV6-57 | MEF2C | 0.32423999 | 1.2551E-10 | 2.7492E-10 |
| IGLV6-57 | POU2F2 | 0.38572439 | 9.4539E-15 | 2.9236E-14 |
| IGLV6-57 | PRDM1 | 0.44210175 | 2.2432E-19 | 1.2508E-18 |
| IGLV6-57 | RUNX3 | 0.36561144 | 2.6506E-13 | 7.4461E-13 |
| IGLV6-57 | SP140 | 0.53121172 | 1.0811E-28 | 1.4209E-27 |
| IGLV6-57 | SPI1 | 0.50005725 | 4.0375E-25 | 4.0157E-24 |
| IGLV6-57 | STAT4 | 0.45515339 | 1.4156E-20 | 9.3027E-20 |
| IGLV6-57 | STAT5A | 0.36968246 | 1.3752E-13 | 3.8929E-13 |
| IGLV6-57 | TRIM22 | 0.4014715 | 5.8965E-16 | 2.0865E-15 |
| IL2RG | CD74 | 0.53903046 | 1.1964E-29 | 1.8345E-28 |
| IL2RG | CIITA | 0.43000765 | 2.6154E-18 | 1.3367E-17 |
| IL2RG | EOMES | 0.42221258 | 1.2098E-17 | 5.936E-17 |
| IL2RG | ETS1 | 0.32369131 | 1.3537E-10 | 2.9478E-10 |
| IL2RG | FOXP3 | 0.50522627 | 1.0929E-25 | 1.2187E-24 |
| IL2RG | GATA3 | 0.33702448 | 2.0597E-11 | 4.8277E-11 |
| IL2RG | IKZF1 | 0.49624021 | 1.0449E-24 | 1.0119E-23 |
| IL2RG | IRF1 | 0.42901986 | 3.1826E-18 | 1.6044E-17 |
| IL2RG | IRF4 | 0.44575728 | 1.0471E-19 | 6.1163E-19 |
| IL2RG | POU2F2 | 0.34032133 | 1.2748E-11 | 3.0662E-11 |
| IL2RG | SP140 | 0.52132857 | 1.6115E-27 | 1.913E-26 |
| IL2RG | SPI1 | 0.48440107 | 1.8509E-23 | 1.5535E-22 |
| IL2RG | SPIB | 0.44020616 | 3.3181E-19 | 1.8225E-18 |
| IL2RG | STAT4 | 0.41609322 | 3.9168E-17 | 1.7159E-16 |
| IL2RG | STAT5A | 0.41337059 | 6.5556E-17 | 2.6511E-16 |
| IL2RG | TRIM22 | 0.46510084 | 1.5891E-21 | 1.1368E-20 |
| IL33 | ETS1 | 0.33315584 | 3.5905E-11 | 8.2068E-11 |
| IL33 | IKZF1 | 0.34500702 | 6.3833E-12 | 1.5872E-11 |
| IL33 | MAF | 0.33631803 | 2.281E-11 | 5.2792E-11 |
| IL33 | MEF2C | 0.55513791 | 1.066E-31 | 2.3076E-30 |
| IL33 | POU2F2 | 0.30445851 | 1.7482E-09 | 3.4963E-09 |
| IL33 | TRIM22 | 0.30426948 | 1.791E-09 | 3.5627E-09 |
| TNFRSF17 | CD74 | 0.48891067 | 6.275E-24 | 5.6322E-23 |
| TNFRSF17 | CIITA | 0.44672492 | 8.5453E-20 | 5.072E-19 |
| TNFRSF17 | EOMES | 0.5020348 | 2.4554E-25 | 2.5817E-24 |
| TNFRSF17 | ETS1 | 0.4168312 | 3.4037E-17 | 1.5091E-16 |
| TNFRSF17 | FOXP3 | 0.53974991 | 9.742E-30 | 1.5587E-28 |
| TNFRSF17 | GATA3 | 0.37785594 | 3.5805E-14 | 1.0626E-13 |
| TNFRSF17 | IKZF1 | 0.63284369 | 2.3532E-43 | 1.0825E-41 |
| TNFRSF17 | IRF1 | 0.31786594 | 2.9956E-10 | 6.2635E-10 |
| TNFRSF17 | IRF4 | 0.82741911 | 1.6963E-95 | 6.2423E-93 |
| TNFRSF17 | MEF2C | 0.4204256 | 1.7092E-17 | 8.1198E-17 |
| TNFRSF17 | POU2F2 | 0.54308751 | 3.7316E-30 | 6.5392E-29 |
| TNFRSF17 | PRDM1 | 0.47167318 | 3.6005E-22 | 2.7604E-21 |
| TNFRSF17 | RUNX3 | 0.39435563 | 2.1042E-15 | 6.8784E-15 |
| TNFRSF17 | SP140 | 0.67195374 | 1.3813E-50 | 1.0166E-48 |
| TNFRSF17 | SPI1 | 0.43591598 | 7.9757E-19 | 4.2537E-18 |
| TNFRSF17 | SPIB | 0.36968322 | 1.375E-13 | 3.8929E-13 |
| TNFRSF17 | STAT4 | 0.5756833 | 1.7598E-34 | 4.9816E-33 |
| TNFRSF17 | TRIM22 | 0.4843862 | 1.8575E-23 | 1.5535E-22 |
